# The complete chloroplast genome sequence of *Pterocarpus macrocarpus*

**DOI:** 10.1080/23802359.2020.1714509

**Published:** 2020-01-20

**Authors:** Jinfeng Zhang, Yunqing Li, Xiaolong Yuan, Yi Wang

**Affiliations:** Laboratory of Forest Plant Cultivation and Utilization, Yunnan Academy of Forestry, Kunming, People’s Republic of China

**Keywords:** *Pterocarpus macrocarpus*, chloroplast, Illumina sequencing, phylogenetic analysis

## Abstract

The first complete chloroplast genome (cpDNA) sequence of *Pterocarpus macrocarpus* was determined from Illumina HiSeq pair-end sequencing data in this study. The cpDNA is 154,734 bp in length, contains a large single copy region (LSC) of 88,418 bp and a small single copy region (SSC) of 18,790 bp, which were separated by a pair of inverted repeats (IR) regions of 23,763 bp. The genome contains 127 genes, including 84 protein-coding genes, 8 ribosomal RNA genes, and 35 transfer RNA genes. The overall GC content of the whole genome is 36.3%, and the corresponding values of the LSC, SSC, and IR regions are 33.8%, 30.2%, and 43.3%, respectively. Further phylogenomic analysis showed that *P. macrocarpus* in Pterocarpus genus clade in Papilionoideae subfamily.

*Pterocarpus macarocarpus* is the species of the subfamily Papilionoideae in Fabaceae, and native to Myanmar, Thailand, Laos, Cambodia and Vietnam (Doungyotha and Owens 2002). The heartwood of *P. macarocarpus* is a kind of expensive reddish hardwood for making furniture and handicrafts (Li 2018). The wood aroma of *P. macarocarpus* can promote human blood circulation and enhancing the body’s immune function (Chen et al. 2018). The homopterocarpin from *P. macarocarpus* showed anti-cancer activity with outstanding performance and cryptomeridiol from *P. macarocarpus* is a natural product with anti-Alzheimer’s disease and antispasmodic properties (Jiang et al. 2018). Macrocarposide from *P. macarocarpus* showed neuroprotective effect (Verma et al. 1986). Therefore, *P. macarocarpus* has huge potential value in timber and biomedicine.

However, there has been no genomic studies on *P. macrocarpus*.

Herein, we reported and characterized the complete *P. macrocarpus* plastid genome. The GenBank accession number is MN823699. One *P. macrocarpus* individual (specimen number: 201907031) was collected from Puwen, Yunnan Province of China (23°31′28″N, 101°37′17″E). The specimen is stored at Yunnan Academy of Forestry Herbarium, Kunming, China and the accession number is ZJFEP120. DNA was extracted from its fresh leaves using DNA Plantzol Reagent (Invitrogen, Carlsbad, CA, USA).

Paired-end reads were sequenced by using Illumina HiSeq system (Illumina, San Diego, CA). In total, about 20.7 million high-quality clean reads were generated with adaptors trimmed. Aligning, assembly, and annotation were conducted by CLC de novo assembler (CLC Bio, Aarhus, Denmark), BLAST, GeSeq (Tillich et al. 2017), and GENEIOUS v 11.0.5 (Biomatters Ltd, Auckland, New Zealand). To confirm the phylogenetic position of *P. macrocarpus*, other twelve species of *Papilionoideae* subfamily from NCBI were aligned using MAFFT v.7 (Katoh and Standley 2013). The Auto algorithm in the MAFFT alignment software was used to align the fifteen complete genome sequences and the G-INS-i algorithm was used to align the partial complex sequences. The maximum likelihood (ML) bootstrap analysis was conducted using RAxML (Stamatakis 2006); bootstrap probability values were calculated from 1000 replicates. *Leucaena trichandra* (KT428297) and *Mimosa pudica* (MH671330) were served as the out-group.

The complete *P. macrocarpus* plastid genome is a circular DNA molecule with the length of 154,734 bp, contains a large single copy region (LSC) of 88,418 bp and a small single copy region (SSC) of 18,790 bp, which were separated by a pair of inverted repeats (IR) regions of 23,763 bp. The overall GC content of the whole genome is 36.3%, and the corresponding values of the LSC, SSC, and IR regions are 33.8%, 30.2%, and 43.3%, respectively. The plastid genome contained 127 genes, including 84 protein-coding genes, 8 ribosomal RNA genes, and 35 transfer RNA genes. Phylogenetic analysis showed that *P. macrocarpus* clustered in Pterocarpus genus clade in *Papilionoideae* subfamily and closed into *Pterocarpus indicus* (Figure 1). The determination of the complete plastid genome sequences provided new molecular data to illuminate the *Papilionoideae* subfamily evolution.

**Figure 1.**
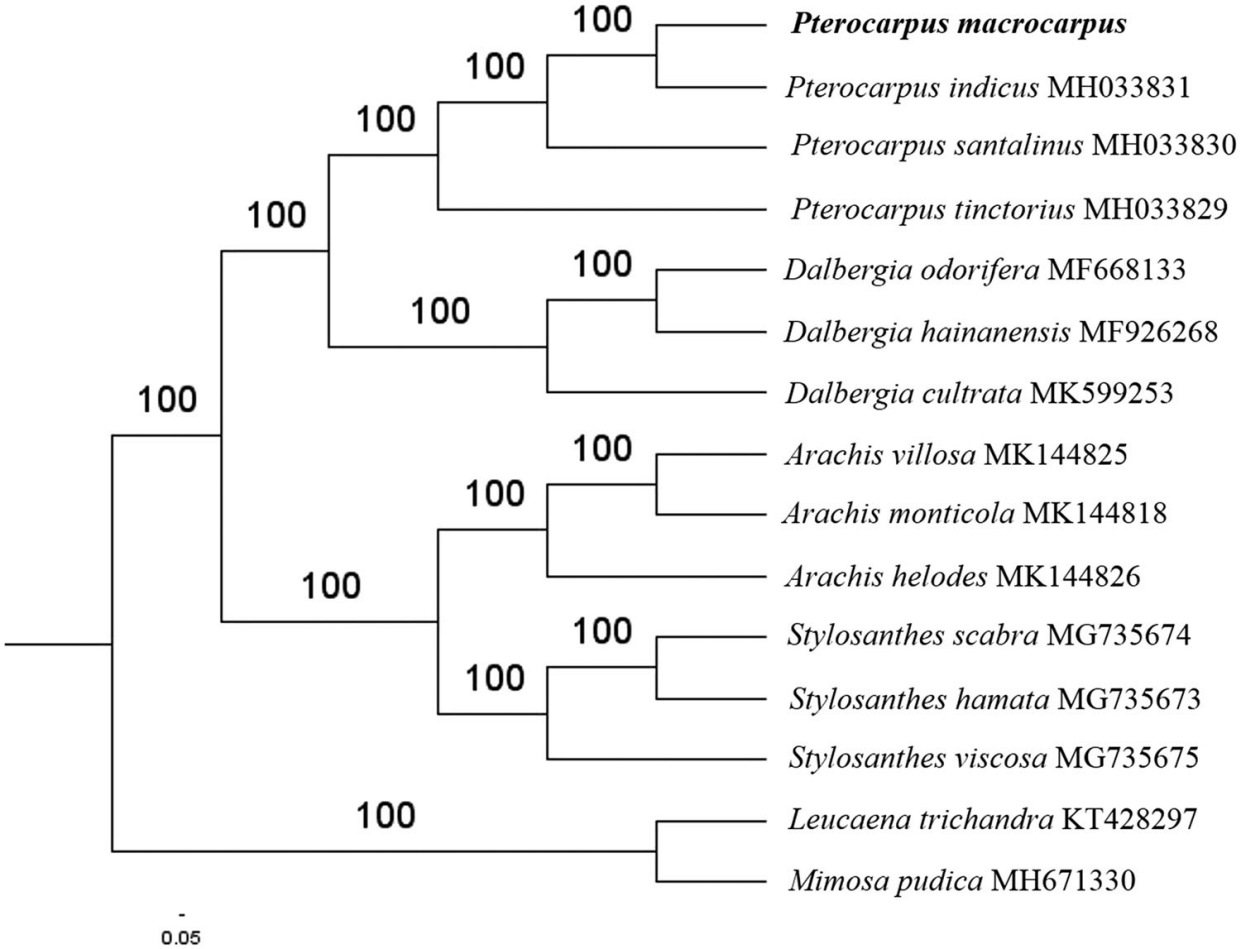
The maximum-likelihood tree based on the thirteen chloroplast genomes of *Papilionoideae* subfamily. The bootstrap value based on 1000 replicates is shown on each node.
